# Nail-Patella Syndrome: A Case Series From Northern India

**DOI:** 10.7759/cureus.47792

**Published:** 2023-10-27

**Authors:** Ganesh S Dharmshaktu, Ishwar S Dharmshaktu

**Affiliations:** 1 Department of Orthopaedics, Government Medical College, Haldwani, Haldwani, IND

**Keywords:** iliac horns, nail patella syndrome, small patella, dystrophic nail, anatomic anomaly

## Abstract

The nail-patella syndrome (NPS) is an uncommon entity with a characteristic set of anomalies. The presence of classical tetrad of hypoplastic or absent fingernails, hypoplastic or absent patellae, bilateral iliac horns and varying grades of elbow deformities are well elucidated in the literature. The spectrum of clinical manifestation varies, resulting in very few cases presenting to the healthcare facility or being diagnosed appropriately. We, hereby, describe our experience of three separate cases of the NPS, diagnosed on clinical and radiological basis. All cases were diagnosed incidentally and none presented to us for consultation regarding the anomalies due to this disorder. In one of the cases, a young girl was managed medically for an associated abdominal complaint. Her father was also found with the clinical features of the disorder thus making the father-daughter duo, part of our series. One case presenting with a femur fracture was managed with fracture fixation surgery leading to an uneventful healing of fracture. There was neither a history of any other family member having similar anomalies nor other systemic disorders in all three cases. Knowledge of the condition may help in improving the diagnosis of NPS and enrich the medical literature.

## Introduction

Nail-patella syndrome (NPS) is a rare disorder, with characteristic anatomical anomalies affecting multiple regions of the body [1]. Classic tetrad of fingernail dysplasia, hypoplastic or absent patellae, presence of iliac horns and elbow deformities are well-described features of NPS [1,2]. The exact ethology is not yet clear but genetic linkage with autosomal dominant behaviour has been reported in the medical literature [2]. This disorder, in the medical literature, is also described as hereditary onycho-osteodysplasia, Fong's disease or Turner-Keiser syndrome [3]. Association with a mutation in the gene LMXB1 on chromosome 9 has been described in the literature, and subsequent heterozygous loss of function of the gene is identified [1,4]. Genetic interplay results in the variability of the clinical presentation of this disorder. Careful clinical assessment, thus, is important to diagnose NPS which then can be followed by relevant radiological investigations.

## Case presentation

Relevant details of our experiences with three cases of NPS are described below. A father-daughter duo makes for the two cases whereas one separate case presented with associated femoral shaft fracture.

Case 1

**Figure 1:**
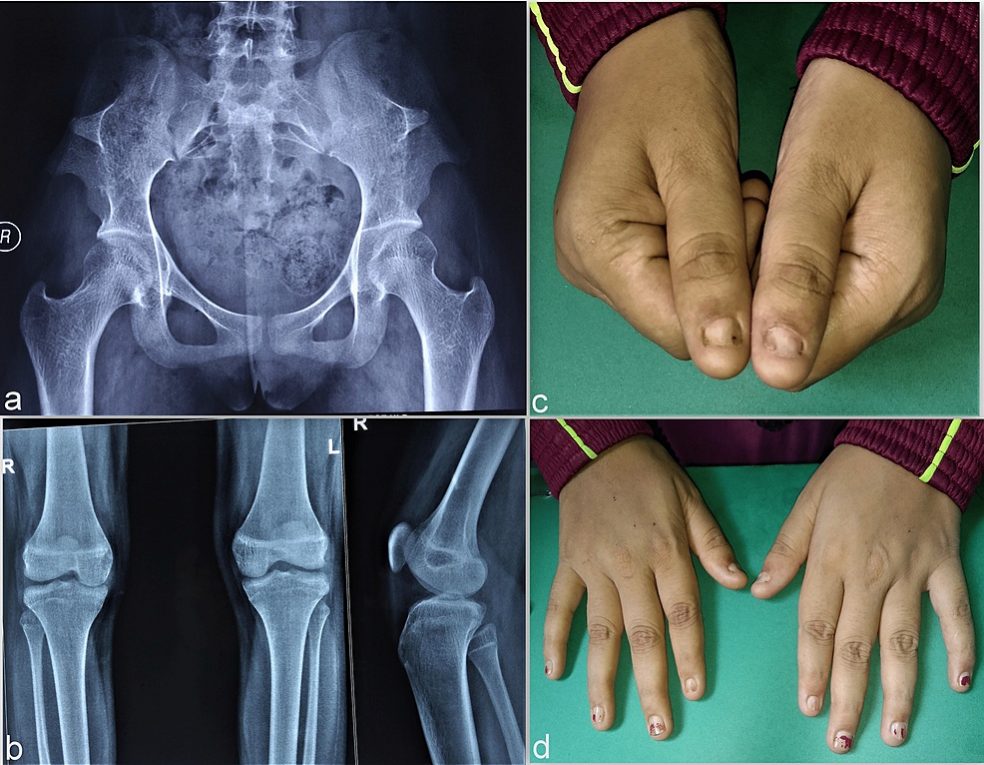
Clinical and radiological features of Case 1 The radiographs showing the presence of bilateral iliac horns in the pelvic region (a). Lateral knee radiograph showing smaller-sized patellae (b). The presence of the hypoplastic changes is more pronounced on bilateral thumbnails (c). Other fingernails show varying grades of hypoplasia and dystrophy (d).

The first two cases were a father-daughter duo, presenting to us when the daughter was brought to the hospital for an episode of abdominal pain. The 14-year-old girl was admitted for the treatment of her abdominal infection in the surgery ward. The abdominal erect radiographs were done as part of the investigation, and she was managed conservatively. Medical management led to gradual recovery and relief from her abdominal complaints. The radiographs were sent for an orthopaedic opinion regarding abnormal bony projections over bilateral iliac bones. These prominences resembled the classic iliac horns, characteristic of the NPS (Figure 1a). After that, clinical evaluation to record other associated described features of NPS in the girl and the accompanying father was initiated. The fingernails of the girl showed varying degrees of hypoplasia, more pronounced in bilateral index and thumbnails (Figures 1b, 1c).

Bilateral patellae were smaller in size but with no clinical problem (Figure 1c). She had no other systemic abnormality and there was no history of similar complaints in her sibling, a younger brother. On the basis of the presence of multiple features, including bilateral nail changes, the presence of bilateral iliac horns and small-sized patellae, a provisional diagnosis of NPS was ascertained. There, however, was no elbow deformity or functional limitation present. She had no clinical problems related to any features of the syndrome and the findings were only incidental. She was, however, asked for periodic review in the future for relevant clinical evaluation.

Case 2

**Figure 2:**
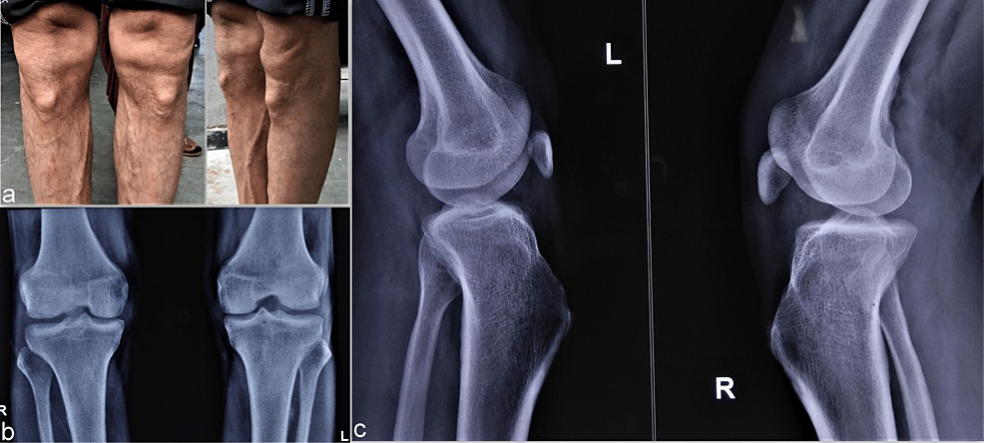
Knee changes in Case 2 The clinical image showing a depressed region at the location of the bilateral patella and a knobby lesion below it, corresponding to bilateral tibial tuberosities (a). The radiograph corresponds to the bilateral hypoplastic patellae (b) and associated hypertrophied bilateral tibial tuberosity (c).

**Figure 3:**
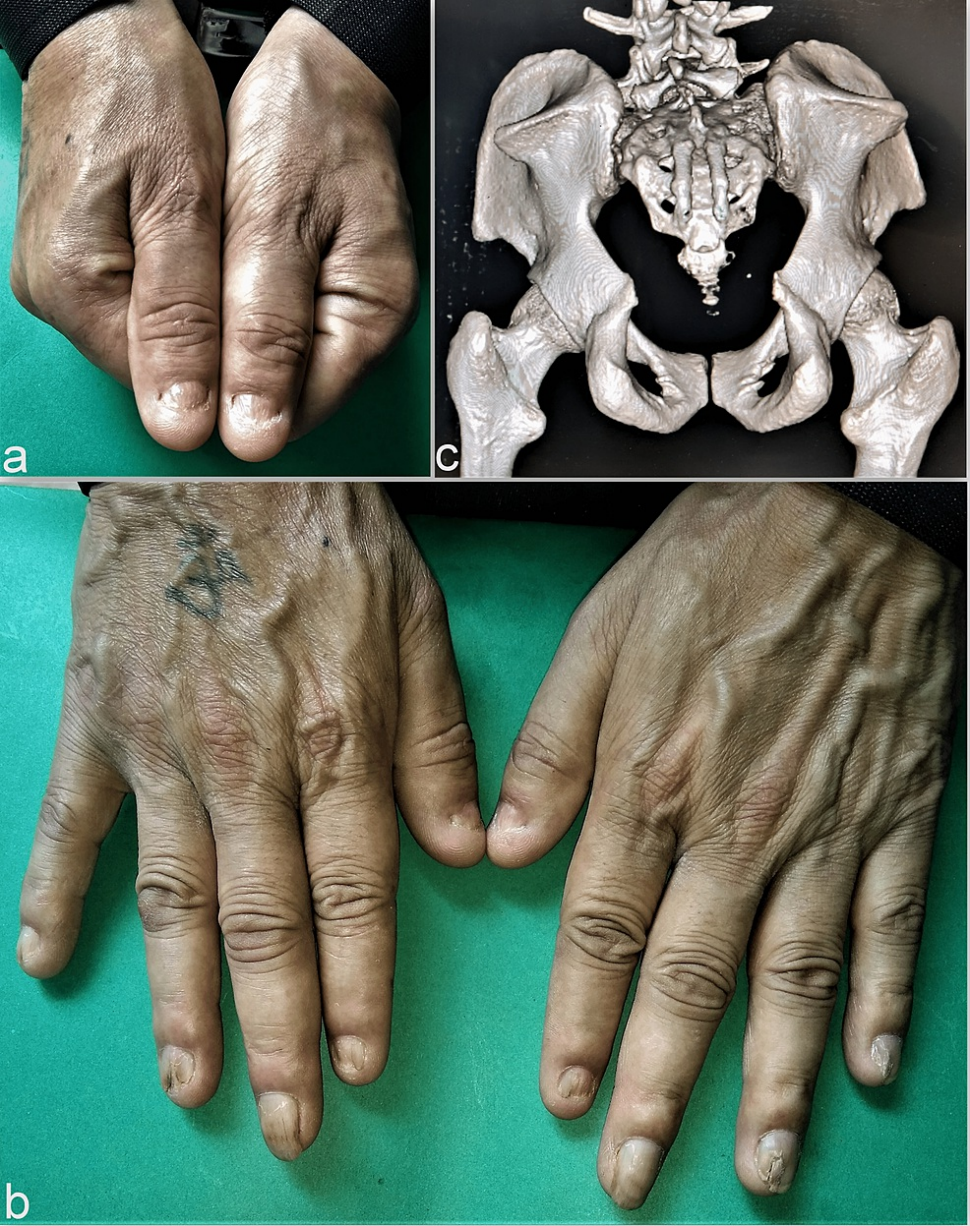
Nail and pelvic anomalies in Case 2 The bilateral thumbnails show hypo-plastic changes (a) in association with multiple deformed fingernails (b). The radiological image shows the presence of the characteristic bilateral iliac horns as posterior projecting structures (c).

The accompanying father (42-year-old) was also clinically evaluated and bilateral small-sized patellae were noted. The patellae were smaller in size as compared to his daughter. There was, however, bilateral resultant hypertrophy of tibial tuberosity (Figure 2a). There was a clinically apparent depression at the sites of patellae representing their smaller sizes (Figures 2b, 2c). Probably, the long-standing patellar tendon strain as a result of smaller patellae, resulted in the hypertrophy of bilateral tibial tuberosities. Knee movement was normal and no similar history in any surviving family members was present. The nail changes were also present in the form of multiple hypoplastic fingernails, also more pronounced in bilateral thumbs (Figures 3a, 3b) like his daughter.

Large bilateral posterior iliac horns were also noted in both radiographs and computed tomography (CT) scan images (Figure 3c).

No other systemic complaints were noted and he refused any further checkups for now. He was, however, asked for a regular follow-up. The consent regarding the use of clinical data was taken from the father along with the assent from the daughter for educational and publication purposes.

Case 3

**Figure 4:**
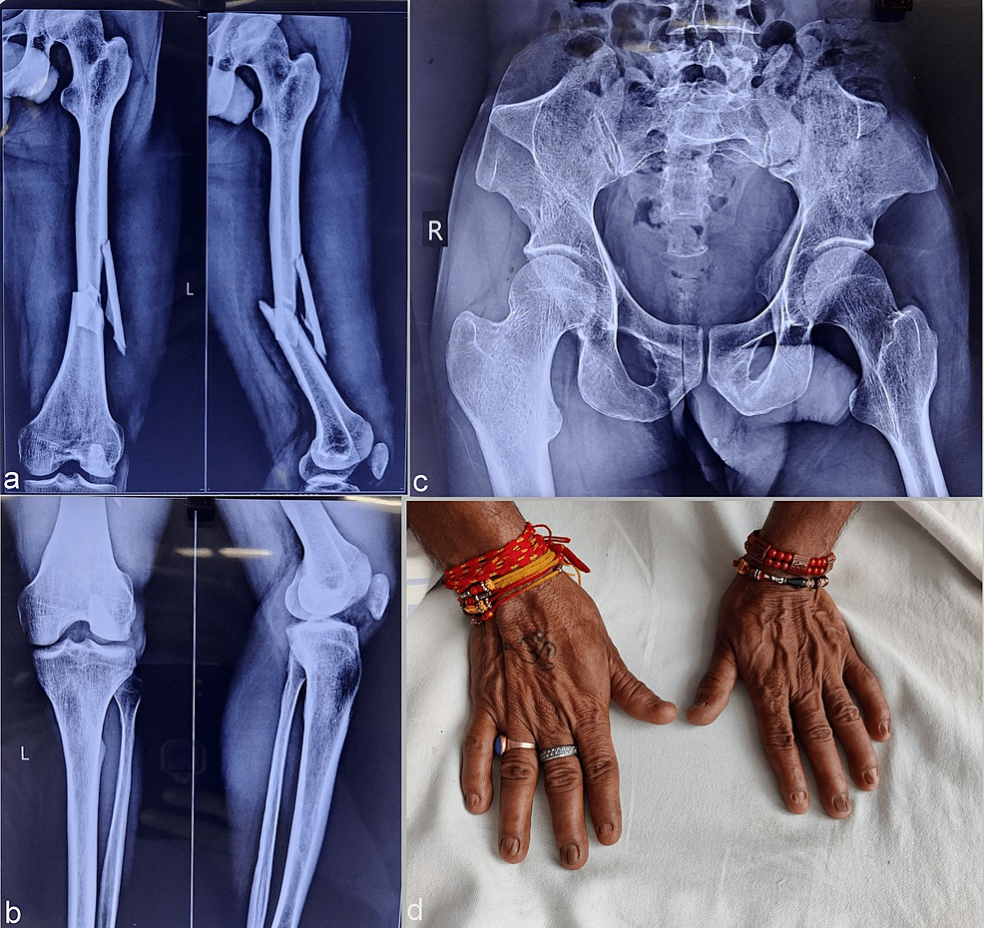
The femoral fracture and associated anomalies of Case 3 The radiographs showing a femur shaft fracture with a large butterfly fragment (a), along with a smaller patella as noted in the associated knee radiographs (b). The pelvic radiograph shows bilateral iliac horns (c). The associated multiple nail changes show deformed and hypoplastic fingernails (d).

A 35-year-old male patient presented to us with a history of road traffic accidents leading to an isolated injury to his left thigh. The injury was closed and the radiograph revealed a femur shaft fracture with a large butterfly fragment (Figure 4a). While the radiographs were done to include both ends of the injured femur, the patella size was noticed to be smaller than what is routinely seen in the normal population (Figure 4b). The clinical patella evaluation showed no subluxation or instability. There was no history of similar complaints in the family. There were exophytic bony projections also noted in the pelvis radiograph. The bony projections, jutting out from the iliac bones, were bilateral and almost similar in shape. These anomalies again resembled the characteristic iliac horns (Figure 4c). The attempt to record other associated conditions was initiated and revealed multiple hypoplastic and deformed multiple fingernails (Figure 4d).

**Figure 5:**
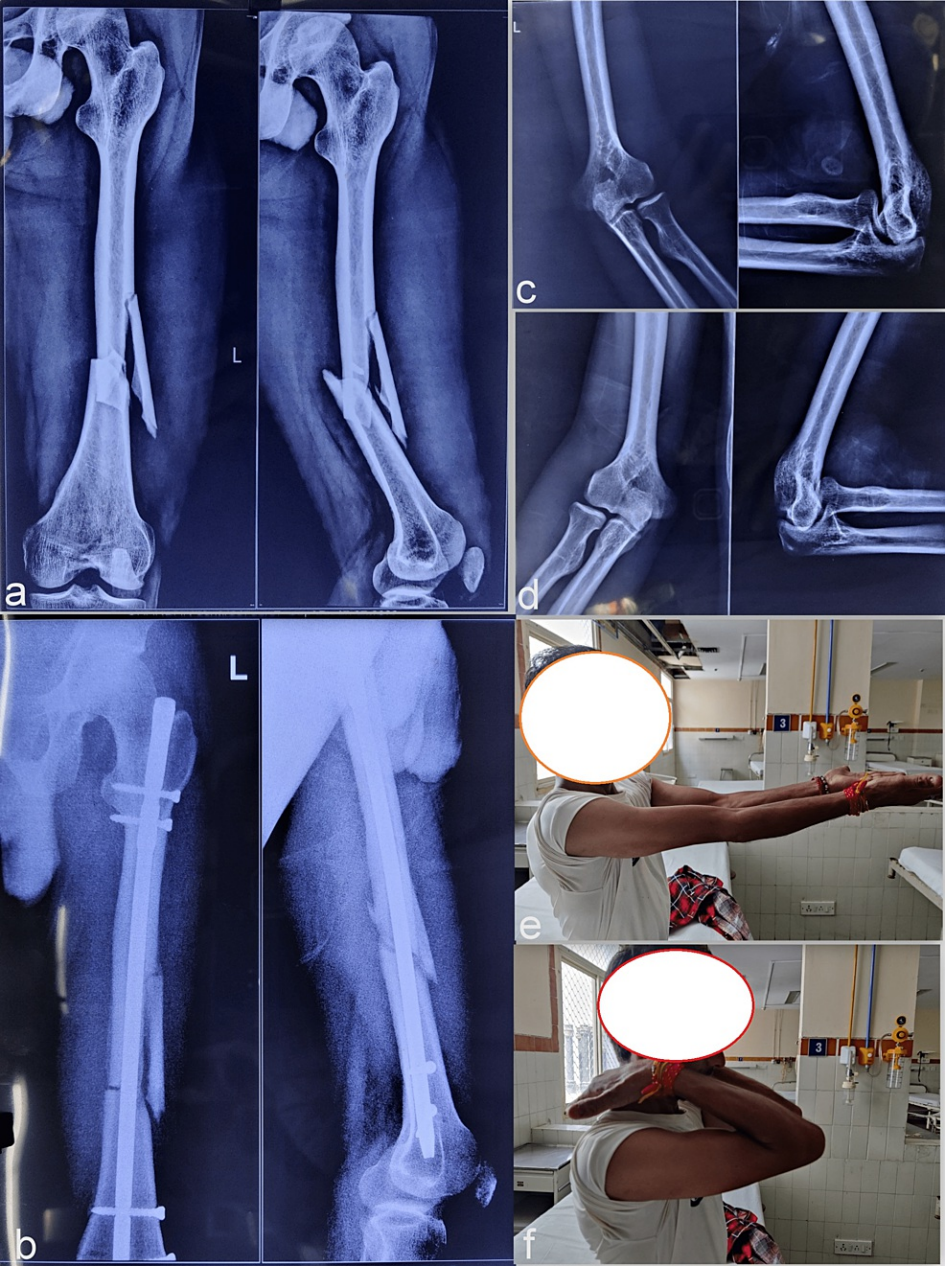
Fracture healing and elbow changes in Case 3 The fracture was managed with closed femur nailing (a) and showed gradual union (b). The elbow radiographs show subtle radial head dysplasia on lateral views (c, d), but without any significant clinical complaints (e, f).

The fracture was treated with closed interlocking femoral nailing under image-intensifier guidance in the standard manner. There were no peri-operative complications or wound issues noted. The fractured butterfly fragment did not attempt to interfere; however, the fragment seemed not completely reduced to the bone (Figure 5a).

The fracture, however, showed a gradual secondary union (Figure 5b). The elbow joint was not affected but abnormal morphological changes in the radial head were noted, underlining subtle bilateral dysplastic changes (Figures 5c, 5d). There was a slight corresponding restriction of terminal extension and pronation-supination movement (Figures 5e, 5f). Regain of pre-injury level of function and painless activities of daily living were noted in the follow-up of nine months.

## Discussion

NPS is an uncommon clinical entity with characteristic clinical features that help to diagnose the condition. Nail and patella changes are very peculiar and knowledge of these characteristic findings may help suspect the condition, even in a busy clinic [5]. Nail changes consist of hypoplasia, dystrophy or absence of one or many nails, more pronounced in fingers. Elbow movement restriction, especially prono-supination and extension, may be another feature [1,3,5]. Elbow movement restriction, especially prono-supination and extension, may be another feature. As many of these cases may accompany some proximal radius anomalies like dysplasia of the radial head, varying grades of restricted pronation-supination movement can be associated. Only in one case, we noted slight terminal restrictions but not having any effect on activities of daily living. Absent or hypoplastic patellae are striking features, which may or may not show associated superolateral subluxation [1,5]. Other associated deformities of the lower femur or knee region should also be recorded in all cases [5,6]. Hypoplastic patellae with lateral subluxation have been reported and anatomical anomalies like sagittal trochlear fibrous septum have been found in one case, the resection of which led to good clinical outcome [7]. In our cases, neither a history of any clinical problem nor any clinical signs of patellar subluxation or knee instability was found. Surgery for patella subluxation of patellar dislocation or instability, if associated with NPS, has been described. Excision of a synovial band or plicae contributing to the subluxation has been highlighted to improve the symptoms [8]. The bone density has also been noted to be lower with subsequent risk of fractures and scoliosis being higher in adults with NPS as compared to the control population [9]. Multi-system involvement has been found and should be sought with relevant evaluation. Renal (30-60%), neurological (neuropathic pains) and ophthalmic elements (glaucoma, ocular hypertension) have been described [1,3,10]. Our cases refused any workup, in the absence of any clinical complaints, but promised periodic assessment in the future.

The life span and outcome are good in cases with NPS. Advanced renal and glaucoma conditions, however, may have serious morbidity in a few affected individuals. Medical management of hypertension and other disorders is advocated and periodic follow-up is warranted. Hypertension and proteinuria are reported as uncommon features in cases with NPS, owing to associated nephropathy in a few cases. Screening shall help in the diagnosis of glaucoma and renal conditions leading to their early management [10]. Being a dominantly inherited familial disorder, the evaluation of other family members should also be done for a detailed assessment of the hereditary pattern [11]. Complex heterogeneity has also been noted in the familial studies of the disorder and in cases with a diagnostic dilemma, establishing the diagnosis with the help of mutation in the LMX1B gene can be done [12]. This investigation, however, is not universally available and feasible. In our resource-limited environment, often high-end investigations are not available and reliance on clinical assessment remains the mainstay. Our series, thus, highlights the importance of knowledge of the condition based on keen clinical observation as an important element of the diagnosis. The knowledge of characteristic deformities may help diagnose the condition appropriately even in busy clinical settings.

## Conclusions

Careful clinical and radiological evaluation is critical to diagnose uncommon conditions. In cases of rare syndromes, the importance of knowledge of the condition and appropriate imaging becomes important. To strengthen the literature, all described clinical features should be documented along with newer features, if any, not described yet. Genetic confirmation, if feasible, can be done for better understanding and future research directions. In the absence of genetic testing, judicious clinical and radiological correlation can be instrumental in the diagnosis. Besides that, any newer clinical feature should also be reported to strengthen the medical literature that will ultimately serve to understand rare conditions more comprehensively.
